# Potential Locations for Non-Invasive Brain Stimulation in Treating Schizophrenia: A Resting-State Functional Connectivity Analysis

**DOI:** 10.3389/fneur.2021.766736

**Published:** 2021-12-15

**Authors:** Yanzhe Ning, Sisi Zheng, Sitong Feng, Binlong Zhang, Hongxiao Jia

**Affiliations:** ^1^The National Clinical Research Center for Mental Disorders & Beijing Key Laboratory of Mental Disorders, Beijing Anding Hospital, Capital Medical University, Beijing, China; ^2^Advanced Innovation Center for Human Brain Protection, Capital Medical University, Beijing, China; ^3^Department of Acupuncture and Moxibustion, Guang'anmen Hospital, China Academy of Chinese Medical Sciences, Beijing, China

**Keywords:** schizophrenia, non-invasive brain stimulation, functional magnetic resonance imaging, functional connectivity, DLPFC (dorsolateral prefrontal cortex)

## Abstract

**Introduction:** Non-invasive brain stimulation (NIBS) techniques have been widely used for the purpose of improving clinical symptoms of schizophrenia. However, the ambiguous stimulation targets may limit the efficacy of NIBS for schizophrenia. Exploring effective stimulation targets may improve the clinical efficacy of NIBS in schizophrenia.

**Methods:** We first conducted a neurosynth-based meta-analysis of 715 functional magnetic resonance imaging studies to identify schizophrenia-related brain regions as regions of interest. Then, we performed the resting-state functional connectivity analysis in 32 patients with first-episode schizophrenia to find brain surface regions correlated with the regions of interest in three pipelines. Finally, the 10–20 system coordinates corresponding to the brain surface regions were considered as potential targets for NIBS.

**Results:** We identified several potential targets of NIBS, including the bilateral dorsal lateral prefrontal cortex, supplementary motor area, bilateral inferior parietal lobule, temporal pole, medial prefrontal cortex, precuneus, superior and middle temporal gyrus, and superior and middle occipital gyrus. Notably, the 10-20 system location of the bilateral dorsal lateral prefrontal cortex was posterior to F3 (F4), not F3 (F4).

**Conclusion:** Conclusively, our findings suggested that the stimulation locations corresponding to these potential targets might help clinicians optimize the application of NIBS therapy in individuals with schizophrenia.

## Introduction

Schizophrenia (SCZ) is a complex psychiatric disorder associated with disturbances in social interaction and communication (1). Despite centuries of research, the pathophysiological cause of SCZ remains elusive. Around 18.8 to 20.8% of the patients with SCZ are thought to be non-responders to antipsychotic drugs (2). It is considered that the efficacious management also requires non-pharmacotherapies to treat SCZ patients in clinic.

Notably, non-invasive brain stimulation (NIBS) techniques have been widely used to improve the clinical symptoms of SCZ. The most commonly used NIBS technique is repetitive transcranial magnetic stimulation (rTMS). Low-frequency (≤1 Hz) rTMS reduces cortical excitability, whereas high-frequency (5–20 Hz) rTMS does the opposite (3). It has been demonstrated that rTMS on the left temporo-parietal region effectively reduced auditory hallucinations than bilateral or sham stimulation (4). Another frequent NIBS application used in treating SCZ is transcranial direct current stimulation (tDCS), which produces polarity-dependent cortical excitability changes (3). The tDCS appeared to be effective not only for ambulatory and higher-functioning patients but also for patients with ultra-treatment resistant SCZ (5). Specifically, NIBS exerts a small transdiagnostic effect on working memory (6). Nonetheless, a meta-analysis indicated that NIBS was not associated with a reliable improvement in working memory for individuals with SCZ (7). A possible reason limiting the efficacy of NIBS for SCZ is the ambiguous stimulation site (8). Indeed, there are several sites reported in studies, such as dorsolateral prefrontal cortex (dlPFC) (9–11), temporoparietal cortex (TPC) (12, 13), and superior temporal gyrus (STG) (14, 15). The sites used in NIBS research were empirical. Thus, it is necessary to identify viable stimulation sites before using NIBS techniques.

Novel stimulation sites for depression (16), mild cognitive impairment (17), and autism (18) were identified by combining meta-analysis and functional connectivity (FC) analysis from three pipelines. The pipelines contain brain surfaces from (1) meta-analysis, (2) FC analysis results from disease network, and (3) FC analysis results from each disease-associated region of interest (ROI). This method combines the preponderance of meta-analysis and the FC analysis. Researchers optimized the sites of NIBS for treating neuropsychiatric disorders, suggesting the worth of a connectivity-based targeting strategy for NIBS techniques. However, there have been no studies using this method to find potential sites for SCZ. In the present study, we performed a meta-analysis and resting-state FC analysis to identify brain surface regions associated with SCZ-related ROIs to investigate potential targets of NIBS treatment in SCZ.

## Patients and Methods

### Patients

A total of 32 right-handed first episode SCZ patients (13 males and 19 females) were included in the FC analysis. The age of these patients was 23.625 ± 7.404, 17 ~ 42 (M ± SD, Min ~ Max) years old. All examinations were carried out under the guidance of the Declaration of Helsinki. The present study was approved by the Ethics Committee of Beijing Anding Hospital, Capital medical university, China. All the subjects were Chinese Han people. Diagnoses were given by two trained psychiatrists using the Mini-International Neuropsychiatric Interview (M.I.N.I.) (19) under DSM-IV criteria. Psychiatric symptomatology was evaluated by using the Positive and Negative Syndrome Scale (PANSS) (20). Participants were excluded if they (a) were < 16 years old, (b) had current comorbid substance-use disorder (daily consumption of substances for at least one year), (c) had a history of neurological disorders or family history of hereditary neurological disorders, (d) had gross morphological anomalies as evidenced by brain MRI scans, and (e) had any electronic or metal implants.

### MRI Data Acquisition

Resting-state functional magnetic resonance imaging (RS-fMRI) data were acquired with a 3.0 Tesla MRI scanner (Prisma 3.0; Siemens, Germany) in the Beijing Anding Hospital, Capital medical university, China. RS-fMRI were acquired with a single-shot, gradient-recalled echo-planar imaging sequence with the following parameters: repetition time = 2000 ms, echo time = 30 ms, flip angle = 90°, matrix = 64 × 64, field of view = 200 mm × 200 mm, slice thickness = 3.5 mm, gap = 1 mm, 33 axial sections, and 240 volumes.

High-resolution brain structural images were acquired with a T1-weighted three-dimensional (3D) multi-echo magnetization-prepared rapid gradient-echo (MPRAGE) sequence [echo time: 3.39 ms, repetition time: 2,530 ms, slice thickness 1.3 mm, voxel size: 1.3 × 1 × 1 mm^3^, field of view (FOV): 256 × 256 mm^2^, and volume number: 128].

Before scanning, all participants were asked to rest for 30 min and were instructed to stay still, keep their eyes closed, and not fall asleep during scanning. Foam head holders were immobilized to minimize head movements during scanning.

### Image Processing

Firstly, the initial five volumes of the RS-fMRI data were removed. Secondly, the subjects whose head motion evaluated by the mean relative root mean square (RMS) exceeded 0.2 mm or whose maximum head motion exceeded 3 mm were excluded from the analysis. The two steps were conducted by Data Processing and Analysis for Brain Imaging (DPABI) version 5.1 (http://rfmri.org/dpabi) (21). Finally, the remaining subjects' images were preprocessed and analyzed in Conn version 18a (https://sites.google.com/view/conn) (22) and SPM12 (using Conn's default preprocessing pipeline).

Conn's default preprocessing pipelines included both functional images' and structural images' preprocessing steps. Functional images were slice-timing corrected, realigned, normalized [3 × 3 × 3 mm^3^ in Montreal Neurological Institute (MNI) space], and smoothed (6 × 6 × 6 mm^3^). The outliers (>3 SD and >0.5 mm) for subsequent scrubbing regression were detected by the Artifact Detection Tool (www.nitrc.org/projects/artifact_detect/). The structural images were segmented into gray matter, white matter (WM), and cerebral spinal fluid (CSF) and normalized (3 × 3 × 3 mm^3^) to MNI space. Then, linear regression using WM and CSF signals (CompCor; 10 components for WM and five components for CSF), linear trend, subject motion (six rotation/translation motion parameters and six first-order temporal derivatives), and outliers (scrubbing) was conducted to remove confounding effects. After that, the residual blood oxygen level dependent (BOLD) time series was band-pass filtered (0.01–0.1 Hz).

### Identifying SCZ-Associated ROIs From Meta-Analysis

In order to identify SCZ-associated ROIs, we conducted a meta-analysis including 715 fMRI studies under the “schizophrenia” term *via* Neurosynth platform (https://neurosynth.org/;accessed 13 August 2020) (23). The complete list of studies can be found in Supplementary Table S1. Neurosynth platform provides two types of meta-analysis results: the uniformity test maps and association test maps. The uniformity test map was used to identify the SCZ-associated brain regions since the uniformity test maps provide information about the consistency of activation for a given process. Association test maps provided information about the relative selectivity with which regions activate in a particular process (23). A false discovery rate (FDR) adjusted *p*-value of 0.01 was applied to produce the uniformity test map. Next, the coordinates with peak z-scores with all clusters larger than 50 voxels were identified by the xjview toolbox (http://www.alivelearn.net/xjview/). Finally, the 6-mm radius spherical masks centered on the specified peak coordinates were exacted by MarsBaR (http://marsbar.sourceforge.net/, version 0.44). Finally, the masks from MarsBaR and the original uniformity test map from Neurosynth were taken the overlap by xjview. The final ROIs only included the voxels from the original uniformity test map.

### FC Analysis

To explore potential brain surface regions of SCZ, we conducted a seed-to-voxel FC analysis by Conn. At the subject level, the residual BOLD time course was extracted from the ROIs, and Pearson's correlation coefficients were computed between ROIs and all other brain voxels. Then, the coefficients were subsequently transformed into z-scores to increase normality. All subject-level seed maps of seed-to-voxel connectivity were included in a one sample *t*-test to get a group-level correlation map.

### Exploring Potential NIBS Locations for SCZ

**Figure 1:**
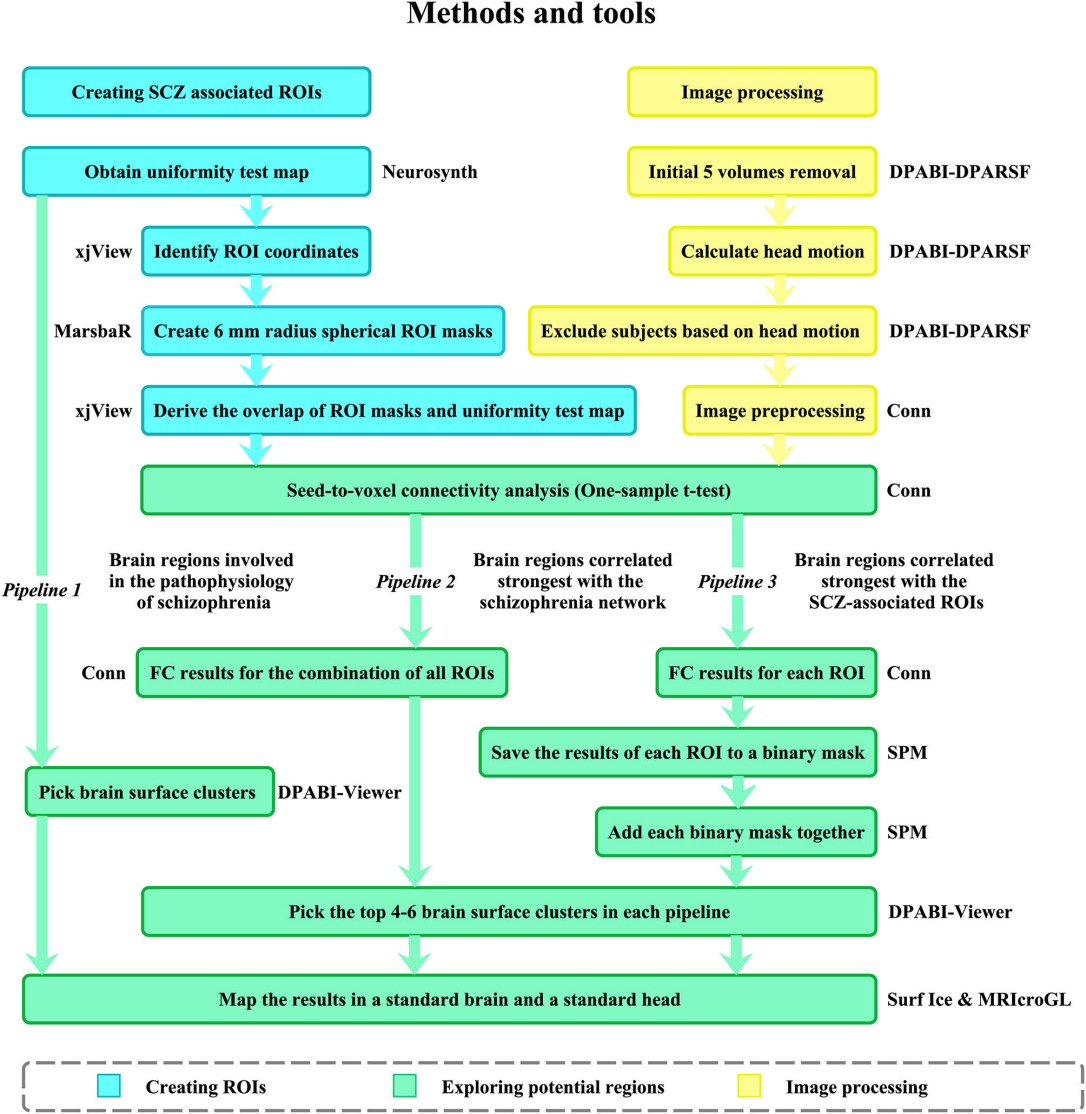
Data processing procedure. SCZ associated ROIs were identified from meta-analysis conducted by Neurosynth. The ROIs were used for FC analysis in 32 SCZ patients. Then, three pipelines were applied to explore potential targets for NIBS in SCZ. ROI, region of interest; FC, functional connectivity; RMS, root mean square; SCZ, schizophrenia.

As the NIBS technique could not access whole brain regions, we used a brain surface mask created in previous studies (16, 18). The mask included the following brain regions: the bilateral pre and post-central gyrus; superior and middle frontal gyrus; superior, inferior, and middle occipital gyrus; superior and inferior parietal lobule; supramarginal gyrus; angular gyrus; superior temporal gyrus; superior temporal pole; middle temporal gyrus (MTG); middle temporal pole; inferior temporal gyrus; opercular inferior frontal gyrus (IFG); Rolandic operculum; triangular IFG; superior medial frontal gyrus; calcarine sulcus; orbital middle, superior and inferior frontal gyri; orbital medial frontal gyrus; supplementary motor area (SMA); paracentral lobule; precuneus; and cuneus. To explore the potential NIBS locations for SCZ, we picked brain surfaces from three different pipelines (Figure 1): (1) meta-analysis; (2) FC analysis results of SCZ network; (3) binary masks combined from each SCZ ROI FC analysis results. In pipelines 2 and 3, a voxel-wise level threshold of *p* < 0.001 and a cluster level family-wise error (FWE) of *p* < 0.05 were applied to obtain group-level correlation maps of ROIs.

#### Pipeline 1 Meta-Analysis

The brain surface clusters were directly picked from the Neurosynth meta-analysis (the uniformity test map) since the brain regions may be directly involved in the pathophysiology of SCZ.

#### Pipeline 2 FC Analysis Results of SCZ Network

The SCZ-associated ROIs were formed into an SCZ network, which was regarded as an ROI for FC analysis in CONN. Next, we excluded the clusters smaller than 20 voxels on the group-level correction map. Finally, four to six surface clusters with the largest peak z-scores were picked, with positive and negative correlation maps, respectively. These clusters represent the brain surface regions possessing the strongest correlations with the SCZ network.

#### Pipeline 3 Combined Binary Masks From FC Analysis Results of Each SCZ-Associated ROI

The group-level correlation map of each SCZ-associated ROI was saved to a binary mask. The binary masks of all ROIs formed a third-level map (positive and negative correlation maps, respectively). The intensity of each voxel in the third-level map represents the number of SCZ–ROIs correlated with the voxel. Finally, we identified four to six surface clusters as potential regions with the largest peak z-scores among all clusters larger than 20 voxels. These clusters represented the brain surface regions which were correlated with the largest number of SCZ-ROIs. The results of the three pipelines were mapped onto a standard brain and a standard head with the international 10–20 system in MNI space (24) using Surf Ice (www.nitrc.org/projects/surfice/) and MRIcroGL (www.mccauslandcenter.sc.edu/mricrogl/).

## Results

### SCZ-Associated ROIs Identified From Meta-Analysis

Fifteen clusters with peak coordinates were identified from the meta-analysis (Table 1). The included studies are listed in the (Supplementary Table S1). These coordinates were used to create 6 mm radius spherical masks, including the bilateral amygdala, insula, thalamus, caudate, and the left caudate, precentral, precuneus, supplementary motor area (SMA), inferior parietal lobule, and the right middle frontal cortex. We took the overlap of these masks and the original meta-analysis map (Supplementary Figure S2). Finally, the refined masks were used for seed-to-voxel connectivity analysis.

**Table 1:** Coordinates of schizophrenia (SCZ) ROIs identified from meta-analysis.

**ClusterID**	**Cluster size**	**T peak**	**Peak coordinates**			**Brain regions**
			**x**	**y**	**z**	
1	188	12.68	−24	−6	−16	Amygdala_L
2	107	10.21	24	−6	−16	Amygdala_R
3	452	20.45	34	26	−4	Insula_R
4	681	20.45	−32	22	−4	Insula_L
5	118	10.91	−10	10	4	Caudate_L
6	149	10.91	14	10	4	Caudate_R
7	81	8.79	6	−14	4	Thalamus_R
8	119	11.62	−10	−16	6	Thalamus_L
9	674	17.27	−46	10	32	Precentral_L
10	97	8.44	40	38	24	Frontal_Mid_R
11	229	13.03	−2	−56	26	Precuneus_L
12	246	14.45	46	8	28	Frontal_Inf_Oper_R
13	722	16.21	0	14	48	SMA_L
14	158	11.62	−30	−56	40	Inferior Parietal_L
15	50	10.21	34	−56	44	Angular_R

*L, left; R, right; SMA, supplementary motor area*.

### Potential NIBS Locations for SCZ

**Figure 2:**
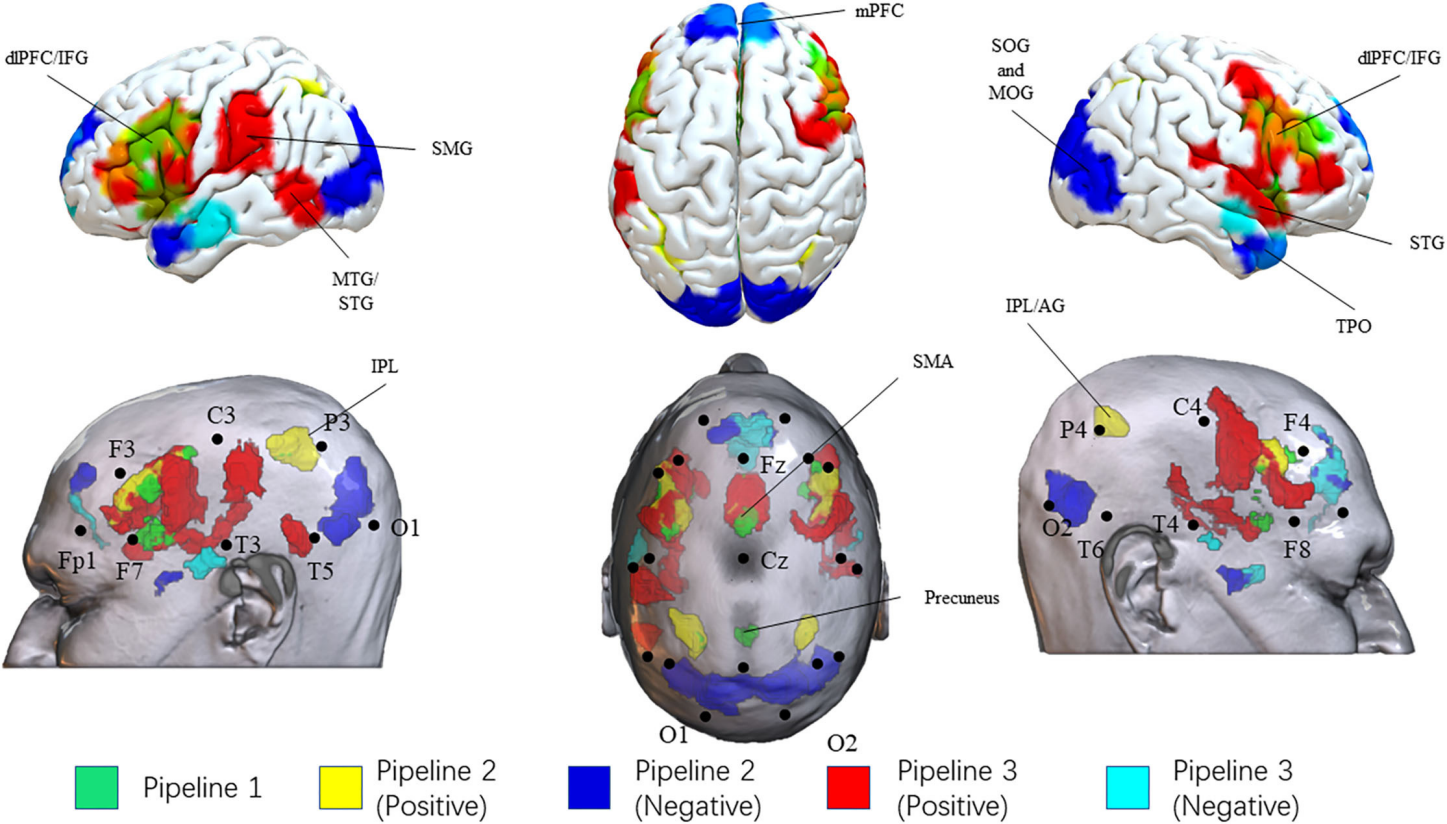
Results of three pipelines. Brain surface regions identified from the three pipelines were presented on the top. Scalp locations corresponding to the brain surface regions were presented on the bottom. Results from pipeline 1, pipeline 2 (positive correlation), pipeline 2 (negative correlation), pipeline 3 (positive correlation), pipeline 3 (negative correlation) were presented as green, yellow, blue, red, and cyan, respectively. L, left; R, right; IPL, Inferior Parietal Lobule; AG, angular gyrus; SMG, supramarginal gyrus SMA, supplementary motor area; dlPFC, dorsolateral prefrontal cortex; IFG, inferior frontal gyrus; mPFC, medial prefrontal cortex; MTG, middle temporal gyrus; STG, superior temporal gyrus; MTG, middle temporal gyrus; TPO, temporal pole; SOG, superior occipital gyrus; MOG, middle occipital gyrus.

Thirty-two SCZ patients were included in this meta-analysis. The results of these three pipelines mapped on a standard brain and a standard head in the MNI space were shown in Table 2 and Figure 2. The original results of each pipeline were in (Supplementary Figures S3–S7).

**Table 2:** Potential locations for non-invasive brain stimulation (NIBS) techniques in SCZ from the three pipelines.

**ID**	**Cluster size**	Peak Intensity^*^	**Peak coordinates**			**Brain regions**	**10–20 system locations**
			**x**	**y**	**z**		
**Pipeline 1**							
1	674	17.2731	−46	10	32	dlPFC/IFG_R	Posterior to F4
2	535	16.2133	0	14	48	SMA	Midpoint to Fz-Cz
3	310	13.3871	−38	22	−6	dlPFC/IFG_L	Posterior to F3
4	246	14.447	46	8	29	dlPFC/IFG_R	
5	158	11.6208	−30	−56	40	IPL_L	P3
6	133	13.0339	−2	−56	26	Precuneus_bilateral	Anterior to Pz
7	97	8.4414	40	38	24	dlPFC/IFG_R	Posterior to F4
8	67	17.2731	34	26	−6	dlPFC/IFG_R	Posterior to F4
9	50	10.2077	34	−56	44	IPL/AG_R	P4
**Pipeline 2 positive**							
1	1,100	19.6838	−42	10	30	dlPFC/IFG_L	Posterior to F3
2	983	17.2673	46	14	30	dlPFC/IFG_R	Posterior to F4
3	529	16.623	−34	−54	48	IPL_L	P3
4	257	18.8981	32	−54	42	IPL_R	P4
5	597	19.5042	6	20	46	SMA	Midpoint to Fz-Cz
**Pipeline 2 negative**							
1	106	−5.3925	52	10	−42	TPO_R	Inferior to T4-F8
2	49	−4.9283	−50	20	−32	TPO_L	Inferior to T3-F7
3	3,888	−8.0841	−16	−90	24	SOG and MOG_bilateral	O1 to O2
4	627	−5.743	−8	62	28	mPFC	Anterior to Fz
5	24	−4.2461	14	58	38	mPFC	Anterior to Fz
**Pipeline 3 positive**							
1	2,724	13	NA	NA	NA	dlPFC/IFG_L	Posterior to F3
2	1,990	15	NA	NA	NA	dlPFC/IFG_R	Posterior to F4
3	857	13	NA	NA	NA	SMA	Midpoint to Fz-Cz
4	605	13	NA	NA	NA	SMG_L	Midpoint to C3-T3
5	439	12	NA	NA	NA	STG_R	Midpoint to F8-T4
6	259	12	NA	NA	NA	MTG/STG_L	Anterior to T5
**Pipeline 3 Negative**							
1	1,227	8	NA	NA	NA	mPFC	Anterior to Fz
2	162	8	NA	NA	NA	MTG_L	Anterior to T3
3	117	8	NA	NA	NA	TPO_R	Inferior to T4-F8
4	39	8	NA	NA	NA	MTG_R	Anterior to T4

* *The intensity of voxels in each pipeline has different meanings. In pipeline 1, it represents z-score from meta-analysis conducted by Neurosynth; in pipeline 2, it represents Z-value from functional connectivity (FC) analysis conducted by Conn; in pipeline 3, it represents the number of SCZ-ROIs correlated with the voxels. Pipeline 3 has no peak intensity because the voxels in each cluster have equal intensity. L, left; R, right; NA, not applicable; IPL, Inferior Parietal Lobule; AG, angular gyrus; SMG, supramarginal gyrus SMA, supplementary motor area; dlPFC, dorsolateral prefrontal cortex; IFG, inferior frontal gyrus; mPFC, medial prefrontal cortex; MTG, middle temporal gyrus; STG, superior temporal gyrus; MTG, middle temporal gyrus; TPO, temporal pole; SOG, superior occipital gyrus; MOG, middle occipital gyrus*.

In pipeline 1, we directly picked up the brain surface regions from the meta-analysis. Finally, the bilateral dlPFC/IFG, SMA, inferior parietal lobule, and precuneus were identified as potential brain surface regions. The 10–20 system coordinates corresponding to the center of these regions were located approximately posterior to F3(F4), midpoint to Fz-Cz, P3(P4), and anterior to Pz. These brain regions may be directly involved in the pathophysiology of SCZ.

In pipeline 2, group-level FC analysis results (SCZ-network as seed) were mapped onto the head surface. Finally, the bilateral dlPFC/IFG (approximately posterior to F3/F4), inferior parietal lobule [IPL (P3/P4)], and SMA (midpoint to Fz-Cz) were found to be positively correlated with the SCZ network. The bilateral temporal pole, superior and middle occipital gyrus, and medial prefrontal cortex were showed overlap negatively correlated with the SCZ network. These regions were located approximately inferior to T4-F8 (T3-F7), midpoint to O1-O2, and anterior to Fz. These brain regions possessed the strongest correlations with the SCZ network.

In pipeline 3, the largest number of brain surface regions from the third-level FC analysis were picked. Finally, we found that the bilateral dlPFC/IFG, SMA, the right STG, the left MTG/STS, and the left supramarginal gyrus were positively correlated with SCZ-associated ROIs, located approximately posterior to F3(F4), midpoint to Fz-Cz, midpoint to F8-T4, anterior to T5, and midpoint to C3-T3. The bilateral MTG, medial prefrontal cortex, and the right temporal pole were potential brain surface regions positively correlated with SCZ-associated ROIs. The 10–20 system coordinates corresponding to the center of these regions were located approximately anterior to T3(T4), anterior to Fz, and inferior to T4-F8. These brain surface regions correlated with the largest number of SCZ-ROIs.

### Previous NIBS Studies in SCZ

We summarized the targets used in the previous NIBS studies in SCZ from several systematic reviews (8, 25–27) in Table 3. The left dlPFC and the left TPJ were the most frequency targets used in the rTMS and tDCS.

**Table 3:** The targets used in prior studies.

**Targets**	**Reference**	**Symptoms**	**effect**
**rTMS**			
TPC_L	(28–33)	AH	No
TPC_L	(34–40)	AH	Yes
TPC_R	(30)	AH	No
TPC_R	(39)	AH	Yes
TC_L/R	(41)	AH	No
TPJ_L	(4, 12)	AH	Yes
TPJ_L	(31)	Negative	No
STG_L	(14)	AH	No
PFC_R	(42)	Total	No
dlPFC_L	(43–45)	Positive	No
dlPFC_L	(43, 45–53)	Negative	Yes
dlPFC_L	(42, 44, 46, 54–56)	Negative	No
dlPFC_R	(47)	Negative	No
**tDCS (Anode/Cathode)**			
dlPFC_L/TPJ_L	(57–62)	Negative	No
dlPFC_L/TPJ_L	(5, 59, 63, 64)	AH	Yes
dlPFC_L/TPJ_L	(63–65)	Negative	Yes
dlPFC_L/TPJ_L	(57, 58, 60–62)	Positive	No
dlPFC_B/TPJ_B	(57)	Negative	Yes
dlPFC_L/ Fp2	(10)	Negative	No
dlPFC_L/Fp2	(66)	Negative	Yes
dlPFC_B/forearms	(67)	Negative	Yes
dlPFC_L/dlPFC_R	(68, 69)	Negative	Yes

*L, left; R, right; B, bilateral; AH, auditory hallucinations; STG, superior temporal gyrus; TC, temporal cortex; TPC, temporoparietal cortex; TPJ, temporoparietal junction; dlPFC, dorsolateral prefrontal cortex; AH, auditory hallucinations; STG, superior temporal gyrus; TC, temporal cortex; TPC, temporoparietal cortex; TPJ, temporoparietal junction; dlPFC, dorsolateral prefrontal cortex*.

## Discussion

In the present study, we attempt to explore potential brain regions and their corresponding scalp locations for NIBS techniques in treating SCZ. We have detected several potential brain regions by combining meta-analysis and FC analysis, which may contribute to improve the clinical efficacy of NIBS in SCZ.

The bilateral dlPFC/IFG and SMA are the most frequent targets for NIBS treatment in SCZ. Previous meta-analyses have demonstrated that high frequency rTMS and tDCS did not have a reliable improvement on treating SCZ by modulating the dlPFC (7, 70, 71). Contrary to previous clinical trials, our results showed that the location of dlPFC was posterior to F3 (F4) rather than exactly F3 (F4). The brain stimulation experiments in healthy subjects have illustrated that posterior to F3 was the optimal location for stimulating the dlPFC (72). Particularly, the Brodmann Area 9 located on the posterior to F4 in the dlPFC was remarkably associated with negative symptom severity (73). These findings suggest that stimulating the posterior to the F3 (F4) rather than the exact F3 (F4) may improve NIBS efficacy for negative symptoms.

In addition, the resting-state hyperperfusion of the SMA was considered as a marker of current catatonia in SCZ (74). Furthermore, the altered gray matter (74) and white matter volume (75) in the SMA were associated with disturbed motor behavior in SCZ. In our results, the SMA was identified in three pipelines suggesting that the SMA could serve as a considerable NIBS stimulation location for treating SCZ patients, especially those with motor abnormalities. The SMA is easily accessible using NIBS, while it is still virtually left to explore in SCZ. After reviewing the literature and registered trials, we have found just one published trial (76) and one ongoing trial — the Overcoming Psychomotor Slowing in Psychosis trial (OCoPS-P, NCT03921450) — for motor abnormalities in patients with SCZ over the SMA. The published trial, conducted by the Sebastian Walther group, has reported that inhibitory stimulation of the SMA might ameliorate psychomotor slowing in psychosis and major depression patients (76). Our data corroborated the ideas of Sebastian Walther, who suggested that NIBS stimulation of the cortical motor areas could be a practical methodology for improving and restoring motor impairment in SCZ (77).

There are other brain regions identified in our study, involving the bilateral IPL, the right temporal pole (TPO), and the medial prefrontal cortex (mPFC). IPL is one of the structures in the sense of agency and the dysconnectivity of the agency network (78), considered as a center of multisensory integration (79). The bilateral IPL, especially the angular gyrus (AG), may be directly involved in the pathophysiology of SCZ and extremely correlate with the SCZ network. A prior study has implicated that the reduction of IPL might be specific for long-term antipsychotic treatment (80). Our findings further supported that IPL could be a possible target for medication development in the future. Moreover, our results provided functional image evidence for the alteration of mPFC and TPO. The altered dopaminergic and GABAergic modulation in the mPFC is involved in SCZ progression (81). Previous meta-analysis including 4,474 individuals with SCZ has reported that only TPO thickness was negatively correlated with age, and cortical volumes at illness onset and progressive volume were declined in the temporal pole in SCZ (82). The right TPO and mPFC may strongly correlate with the SCZ network and correlate with the largest number of SCZ-associated ROIs. Despite the importance of mPFC and TPO, there remains a paucity of evidence for NIBS techniques treating SCZ over mPFC or TPO.

Interestingly, the dlPFC, SMA, and MTG are the components of the task positive network (TPN), which associates with externally oriented attention (83, 84). The mPFC, IPL, and precuneus play an important role in the default mode network (DMN) related to introspectively oriented cognitive processes, such as self-referential and reflective activity. Consistent with another study (85), we observed that TPN and DMN networks were anti-correlated. Here, our study provided the evidence to support that the anti-correlated networks were relevant to SCZ. Balancing the TPN and DMN network may have a beneficious effect in treating SCZ by NIBS to regulate neural circuits (3, 86).

Some limitations are needed to pay attention in the present study. First, excitatory or inhibitory brain regions in our study are not to be discriminated, which is essential to some NIBS techniques such as TMS. Second, the parameters used in each NIBS technique have not been studied, which may affect the efficacy. Finally, neurosynth-based meta-analysis is not flawless – potential error could occur – although several supporting analyses have been conducted to confirm the validity and sensitivity.

## Conclusion

Combining meta-analysis and FC analysis from three pipelines, we identified several potential NIBS targets on the brain surface (with dlPFC and SMA to be the most promising regions) and locations on the scalp for treating SCZ patients. Specifically, the location of dlPFC was suggested to be posterior to F3 (F4) and not F3 (F4). Besides, we also identified that balancing the TPN and DMN might be a potential strategy to treat SCZ. These identified targets could contribute to improving the efficacy of NIBS in treating SCZ patients.

## Data Availability Statement

The original contributions presented in the study are included in the article/Supplementary Material, further inquiries can be directed to the corresponding author/s.

## Ethics Statement

The studies involving human participants were reviewed and approved by the Ethics Committee of Beijing Anding Hospital. The patients/participants provided their written informed consent to participate in this study.

## Author Contributions

HJ provided his expertise in schizophrenia, managed the data collection, and contributed to the writing of the manuscript. BZ conceived the idea and methodology for the study, designed the study, and contributed to the writing of the manuscript. YN and SZ managed data analyses and wrote the manuscript. SF was contributed to the writing of the manuscript and the graph display. All authors contributed to the article and approved the submitted version.

## Funding

This study was supported by Beijing Natural Science Foundation (Grant No. 7212050), Capital's Funds for Health Improvement and Research (Grant Nos. 2018-1-2122, 2020-4-2126), Beijing Hospitals Authority Clinical Medicine Development of Special Funding (Grant No. ZYLX202129), Beijing Hospitals Authority's Ascent Plan (Grant No. DFL20191901), Beijing Hospitals Authority Youth Program (Grant No. QML20201901), Talents Training Fund of Beijing (Grant No. 2018000021469G292), China Academy of Chinese Medical Sciences Fund for Excellent Young Scholars (Grant No. ZZ14-YQ-017), and China Academy of Chinese Medical Sciences Innovation Fund (Grant No. CI2021A03316).

## Conflict of Interest

The authors declare that the research was conducted in the absence of any commercial or financial relationships that could be construed as a potential conflict of interest. The handling editor declared a past collaboration with one of the authors BZ.

## Publisher's Note

All claims expressed in this article are solely those of the authors and do not necessarily represent those of their affiliated organizations, or those of the publisher, the editors and the reviewers. Any product that may be evaluated in this article, or claim that may be made by its manufacturer, is not guaranteed or endorsed by the publisher.
