# Supplementary figures and images for: Potential Locations for Non-Invasive Brain Stimulation in Treating Schizophrenia: A Resting-State Functional Connectivity Analysis
Source: Front Neurol. 2021 Dec 15;12:766736. doi: 10.3389/fneur.2021.766736 (PMC8715096; doi:10.3389/fneur.2021.766736)

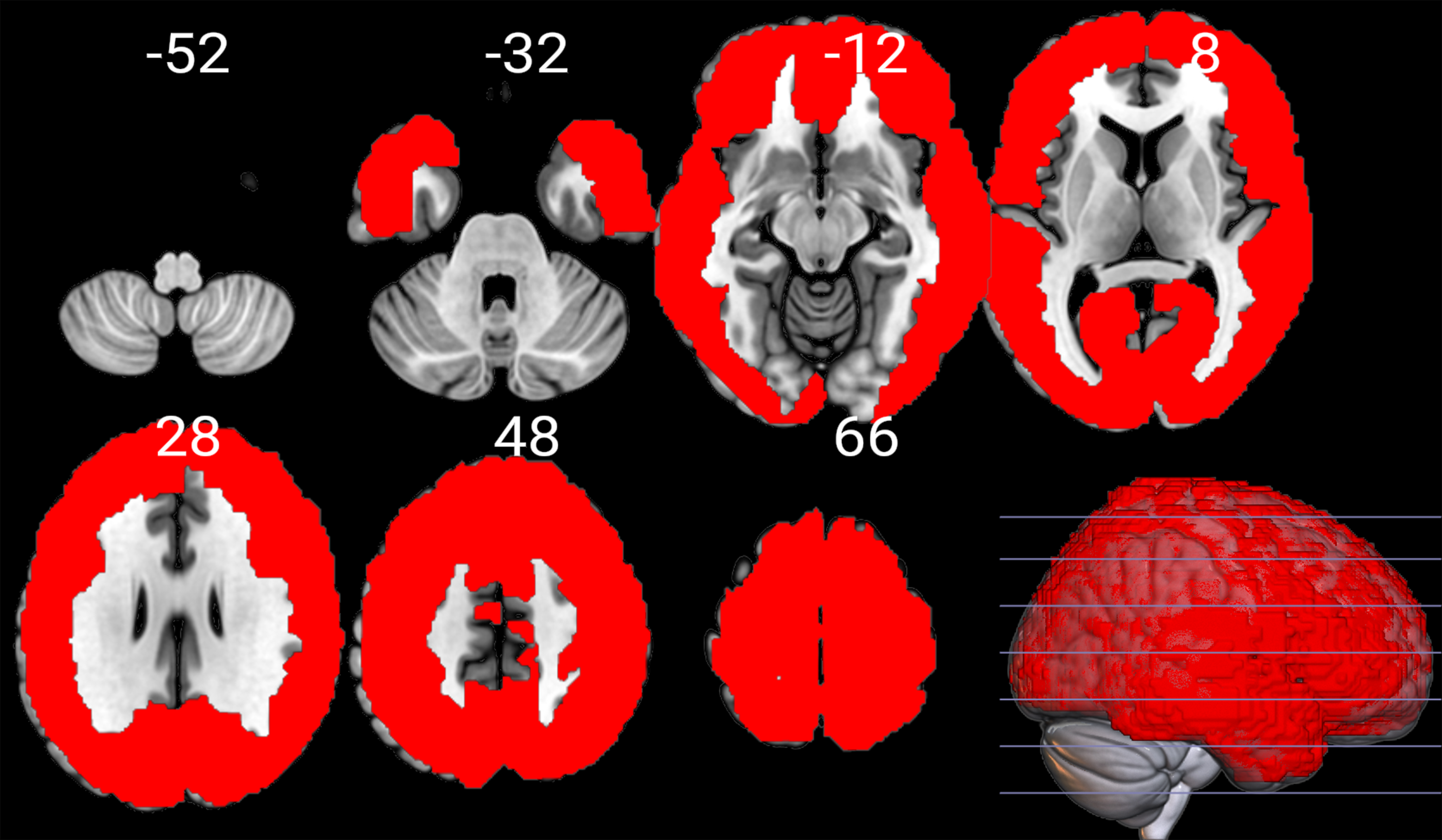

Supplement: Supplementary file 2 [file Image_1.TIF]

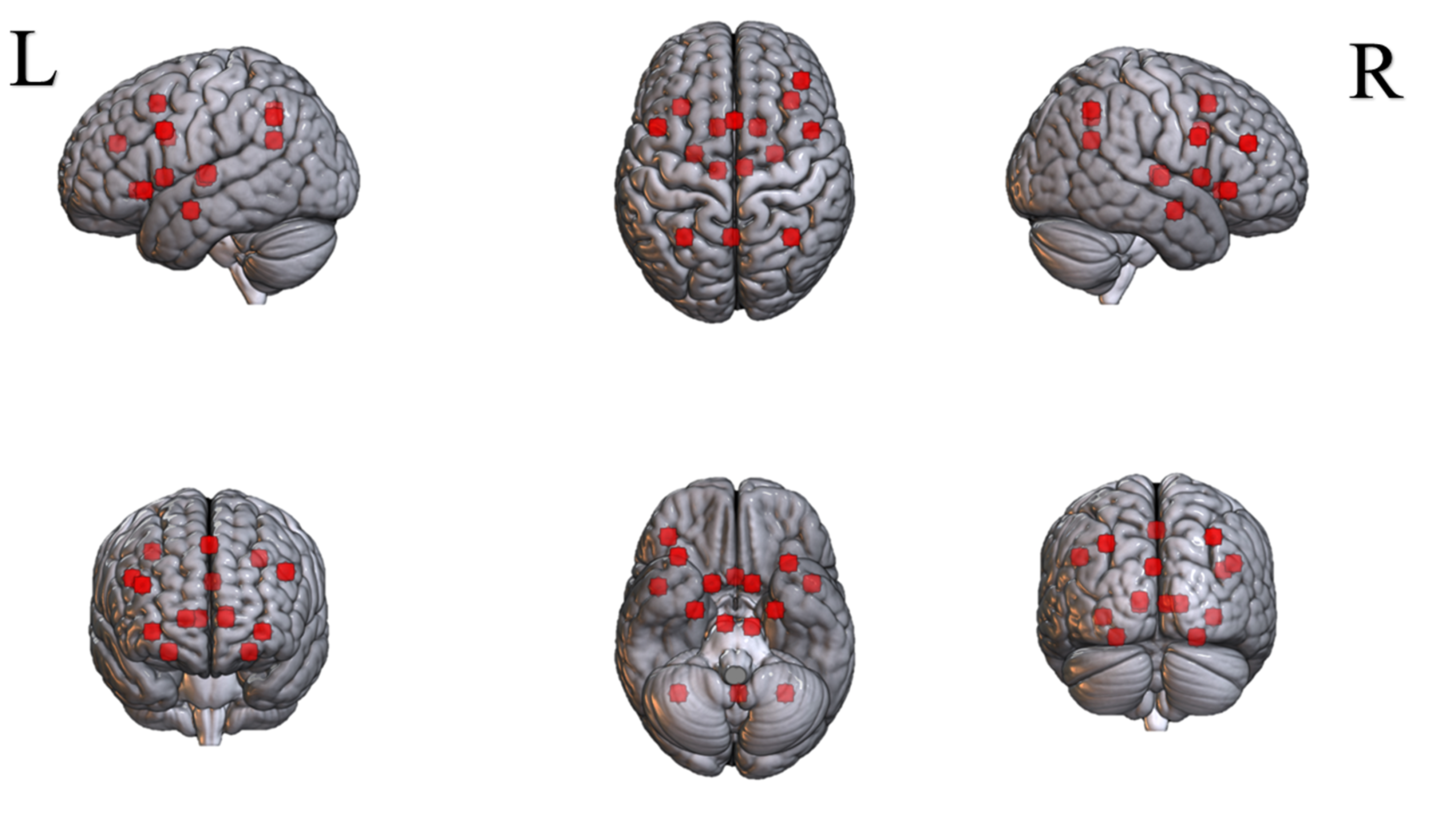

Supplement: Supplementary file 3 [file Image_2.TIF]

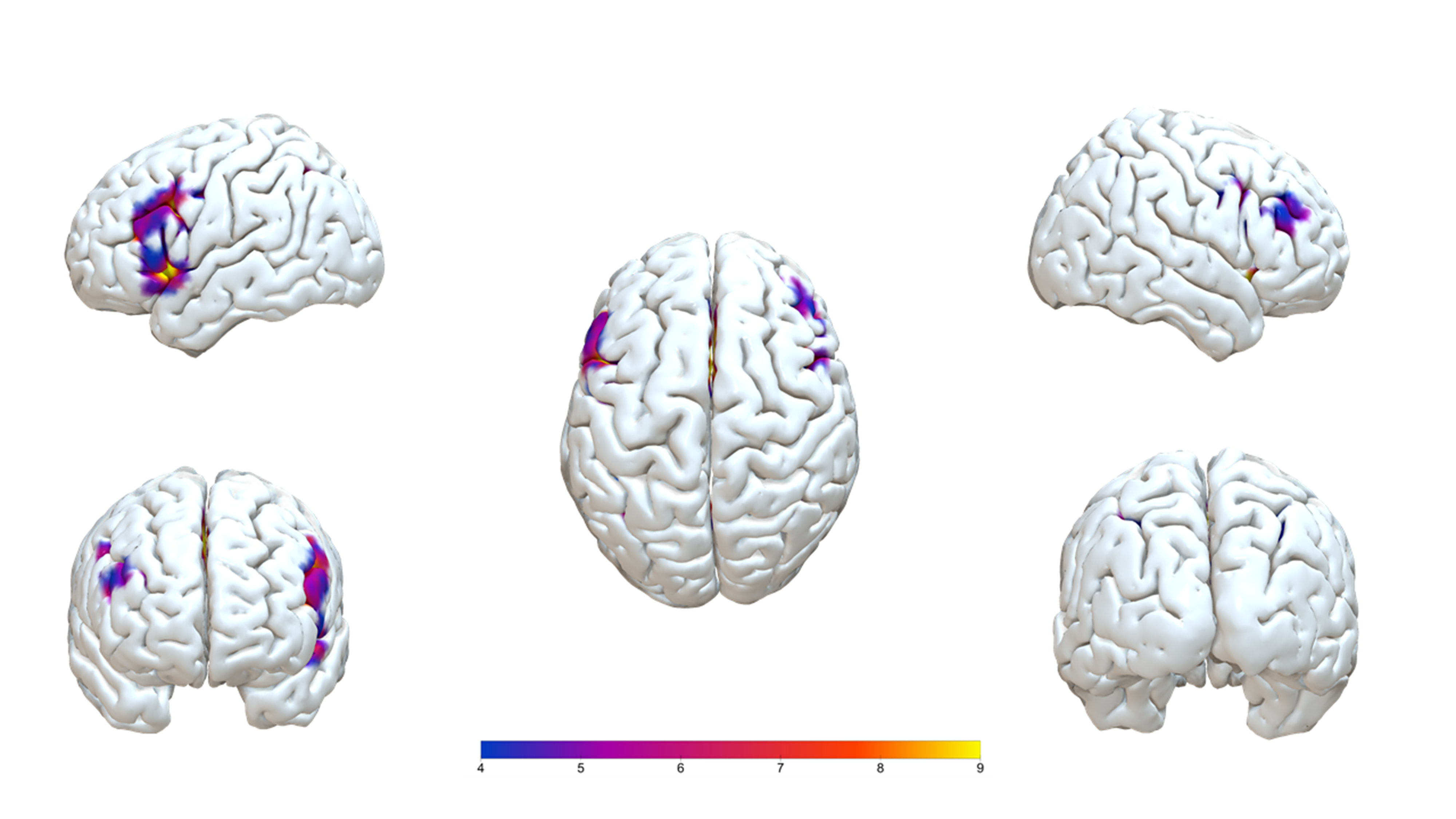

Supplement: Supplementary file 4 [file Image_3.TIF]

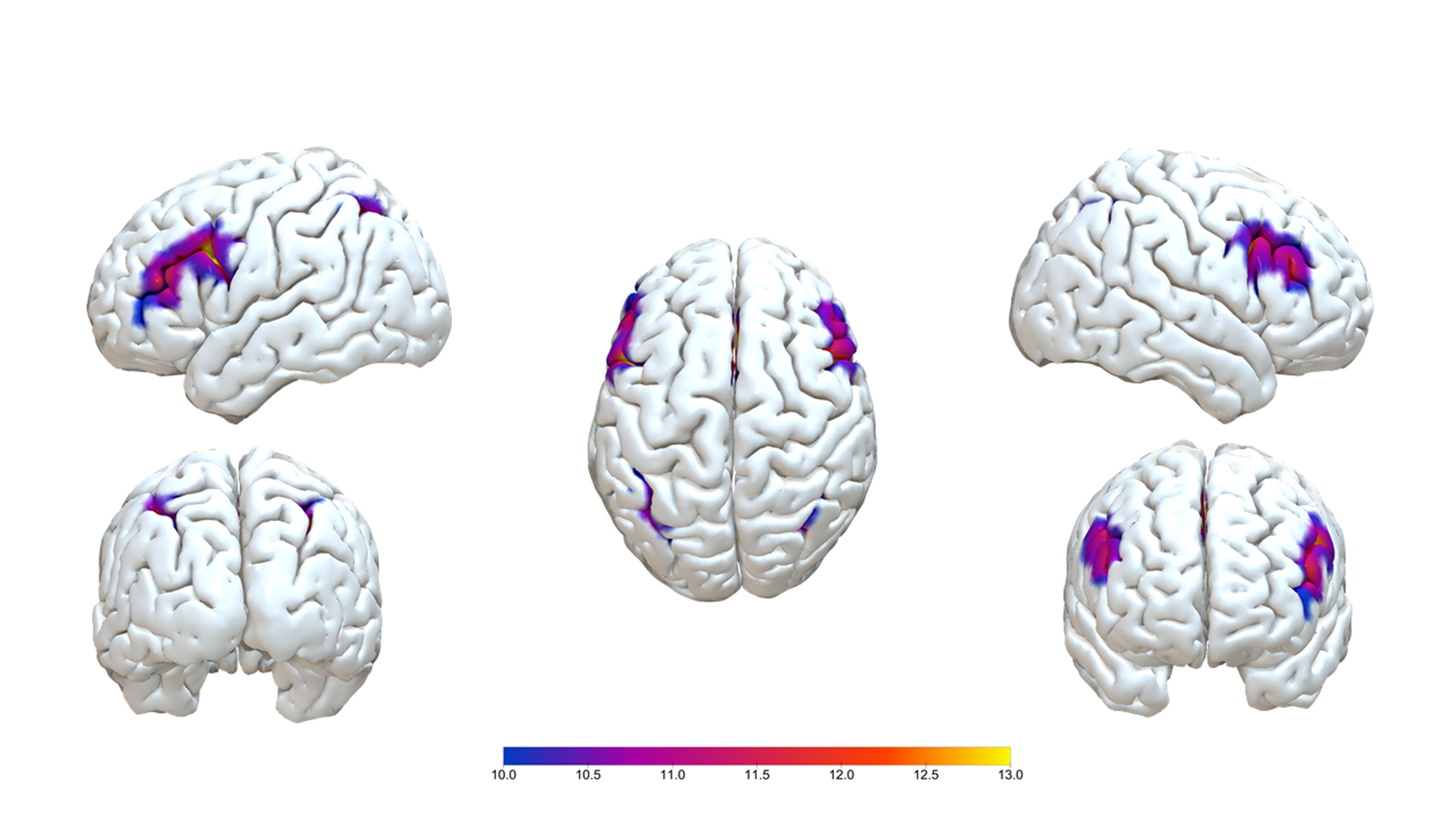

Supplement: Supplementary file 5 [file Image_4.TIF]

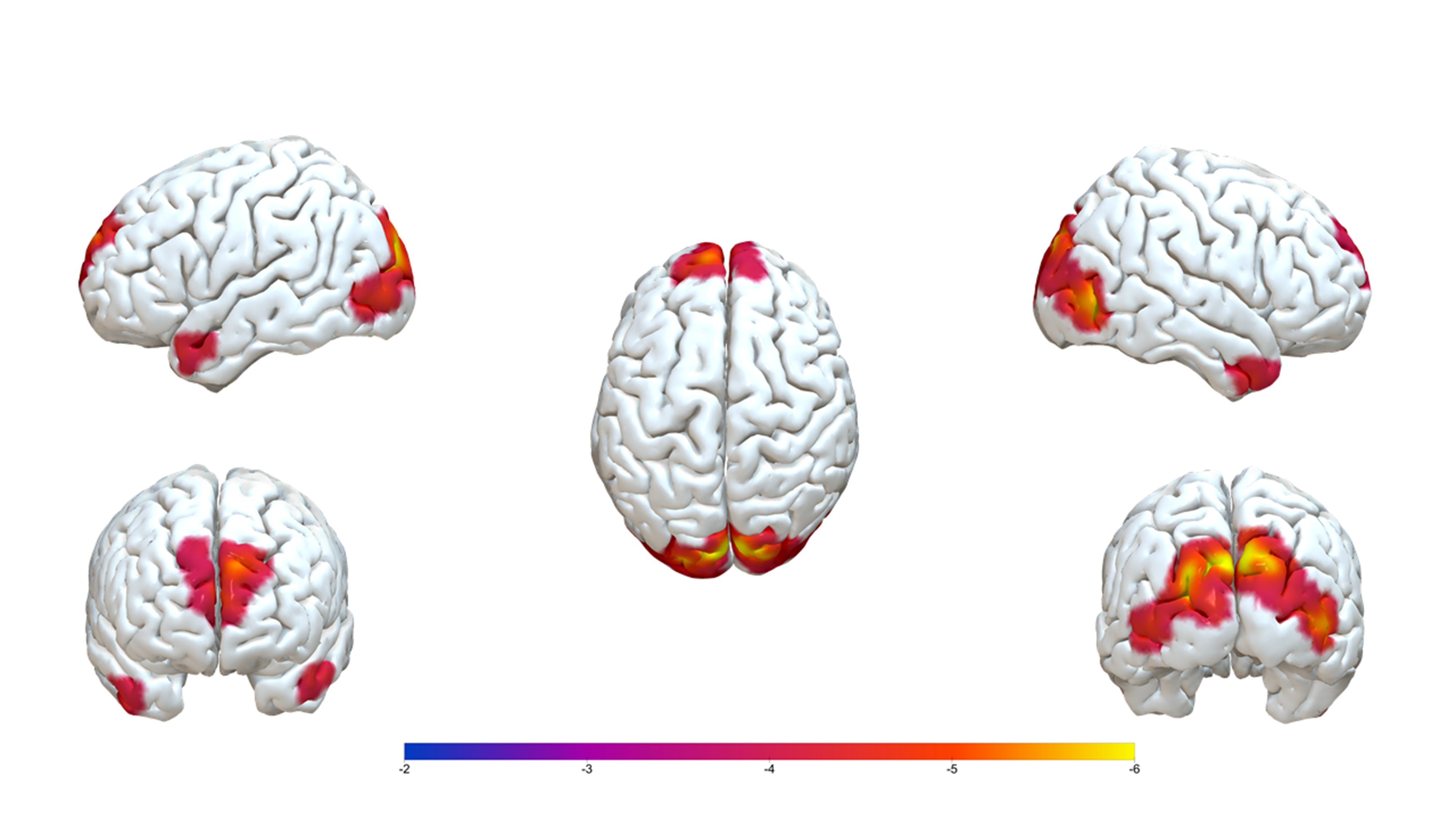

Supplement: Supplementary file 6 [file Image_5.TIF]

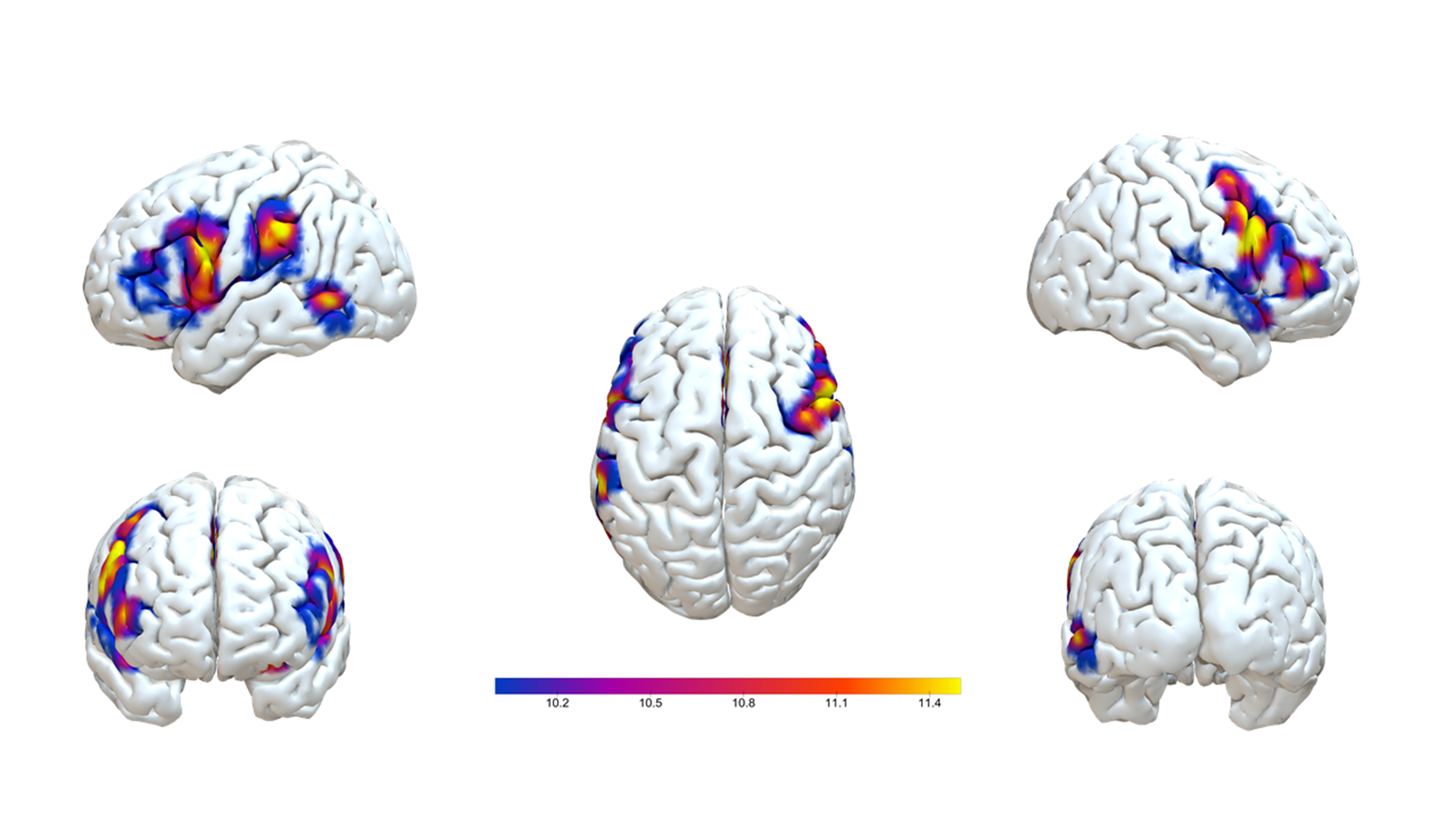

Supplement: Supplementary file 7 [file Image_6.TIF]

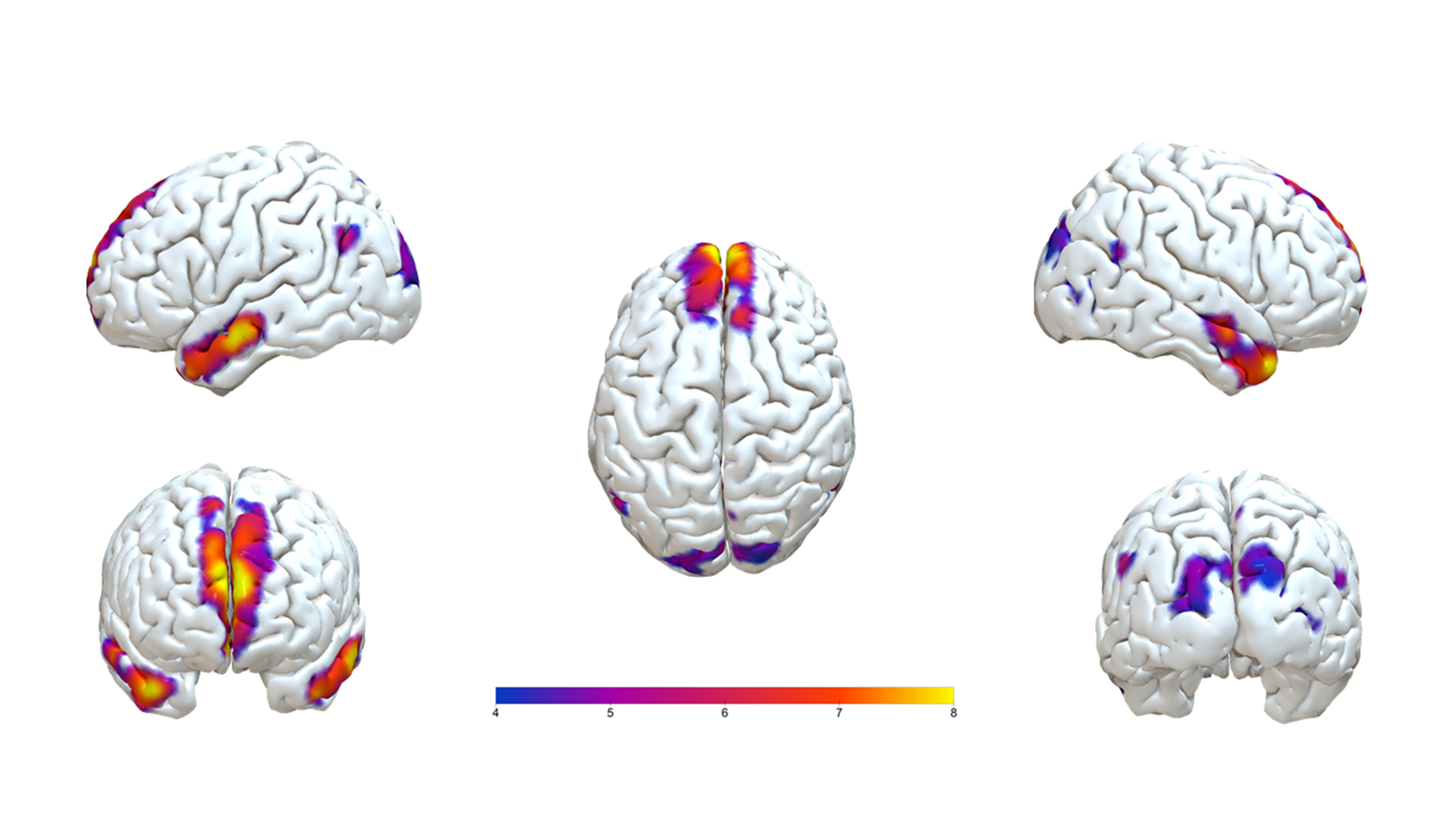

Supplement: Supplementary file 8 [file Image_7.TIF]
